# 5-Hydroxylysine Captures the Suicidally-Inactivated Conformational State of Lysine 5,6-Aminomutase

**DOI:** 10.3390/ijms26178561

**Published:** 2025-09-03

**Authors:** Amarendra Nath Maity, Jun-Ru Chen, Ting-Xi Ke, Shyue-Chu Ke

**Affiliations:** 1 Department of Physics, National Dong Hwa University, Hualien 97401, Taiwan; anmaity@gmail.com (A.N.M.); junru2000@gmail.com (J.-R.C.); 2 Department of Occupational Safety and Health, Chang Jung Christian University, Tainan 711301, Taiwan; ketingxik520520520@gmail.com

**Keywords:** density functional calculations, EPR spectroscopy, hidden conformation, hydroxylysine, radical enzymes

## Abstract

The ability of enzymes to access various conformational states is often essential for their catalytic activity. Lysine 5,6-aminomutase (5,6-LAM), a pyridoxal 5′-phosphate (PLP) and 5′-deoxyadenosylcobalamin (dAdoCbl)dependent enzyme, catalyzes 1,2-amino shift in lysine isomers by shuttling between an open conformational state and a closed conformational state. Nevertheless, suicide inactivation of 5,6-LAM is an obstacle to the realization of its potential as a biocatalyst. In this work, the fate of the reaction of 5-hydroxylysine, an analogue of lysine, is investigated using spectroscopic and computational methods. Although 5-hydroxylysine does not afford any product, results obtained from UV–visible and electron paramagnetic resonance (EPR) spectroscopies demonstrate that initial steps of the catalytic cycle are performed with it. Simulation of the weakly spin-coupled spectrum estimates an intermediate distance between the PLP substrate-based radical and Co(II) in comparison to the that in the open state and the closed state. This distinct conformational state, different from the open state and the closed state, is alluded to in its putative role in suicide inactivation and denoted as the suicidally-inactivated state. Our findings highlight the emergence of EPR spectroscopy as a powerful tool to uncover the hidden conformations in radical enzymes. These results provide new insights into the suicide inactivation of dAdoCbl-dependent enzymes.

## 1. Introduction

Biocatalysis has increasingly been employed in achieving challenging chemical transformations during chemical synthesis of complex molecules [1]. New biocatalysts are being discovered and engineered at a faster pace to add to the existing range of biocatalysts. Lysine 5,6-aminomutase (5,6-LAM) is a promising candidate for biocatalytic application as it has broad substrate and cofactor permissiveness [2,3,4]. It can turn over D-lysine, L-β-lysine, and L-lysine using pyridoxal-5′-phosphate (PLP) and coenzyme B_12_ (5′-deoxyadenosylcobalamin, dAdoCbl) as cofactors. Moreover, it can turn over D-lysine using PLP-N-oxide (PLP-NO), a PLP analogue, instead of PLP as the cofactor [3], while 1-deazaPLP acts as an inhibitor despite emulating the initial few steps [5]. 5,6-LAM follows a radical mechanism initiated by dAdoCbl and undergoes a large-scale conformational movement to shuttle between open and close states. The substrate-free restive, open state positions PLP and dAdoCbl~25 Å apart [6], while a separation of only 5.5 Å between the PLP-substrate analogue-based radical centre and Co(II) of dAdoCbl corresponds to the catalytic close state [7,8]. The open state distance was obtained from X-ray crystallography, while the closed state distance was obtained by employing electron paramagnetic resonance (EPR) spectroscopy. EPR spectroscopy, along with electron-nuclear double resonance (ENDOR) spectroscopy, is one of the most powerful techniques to unravel the intricacies of radical enzymes by taking snapshots of them while in action [9,10]. This is usually achieved by analyzing the EPR spectra obtained by freeze-trapping of transient paramagnetic intermediates. An accurate interspin distance can be estimated by simulating the spin-coupled EPR spectra of the radical pair generated in the reaction of dAdoCbl-dependent enzymes [11]. A significant change in the interspin distance implies the motion of the paramagnetic species. This motion is usually orchestrated by large-scale conformational movement in dAdoCbl-dependent enzymes, resulting in the presence of distinct conformational states [12,13,14,15,16,17]. This feature of possessing distinct thermally accessible conformations is prevalent in enzymes. Hidden conformations were found to be essential for catalysis in proline isomerase [18]. Hidden states of β-lactamase present in pathogenic Gram-negative bacteria have been linked to antibiotic resistance [19]. The activity of monoamine oxidase from Aspergillus niger (MAO-N) was found to be regulated by populating the hidden conformational states as a result of distal mutations [20]. As hidden conformations are inaccessible by X-ray crystallography, techniques such as advanced NMR spectroscopy, single-molecule FRET measurements [21], and ion mobility spectrometry-mass spectrometry (IMS-MS) have been used to uncover hidden conformations [22]. Conformational dynamics of enzymes often plays a key role not only in catalysis but also in various enzymatic events such as enzyme promiscuity and inactivation [23]. A salient aspect of 5,6-LAM is that it undergoes suicide inactivation, which has not been fully understood to date [24]. Similar suicide inactivation was also observed in the cases of other dAdoCbl-dependent enzymes [25,26,27]. Suicide inactivation is one of the impediments towards successful biocatalytic applications. Therefore, understanding the mechanism of suicide inactivation of 5,6-LAM in detail is imperative in order to find a way to suppress it so that the industrial application of 5,6-LAM is realized. Interestingly, ornithine 4,5-aminomutase [14], lysine 2,3-aminomutase [28], and glutamate 2,3-aminomutase [29,30] are closely related enzymes which perform similar 1,2-amino shifts following similar mechanisms [2,31,32] involving 5′-deoxyadenosyl radical (dAdo^•^) [33] and PLP. After being elusive for decades, dAdo^•^ was directly observed and characterized recently in radical *S*-adenosyl methionine enzymes [34,35,36].

The unusual flexibility of 5,6-LAM, as mentioned earlier, led us to explore to further the substrate scope of 5,6-LAM using an analogue of lysine having a substitution on C5. 5-hydroxylysine is a naturally occurring analogue of lysine. In a recent study, Sin and coworkers identified over 1600 5-hydroxylysine sites on 630 proteins in human cells using a constitutional isomer selective chemical proteomic strategy [37]. 5-hydroxylysine has attracted growing research interest over the last decade [38] due to its presence in proteins such as collagen [39] related to rheumatoid arthritis disease, adiponectin that contributes to insulin-sensitizing activity [40], and histone [41] having implications in epigenetic regulation of gene transcription or chromosomal rearrangement. 5-hydroxylysine is a key intermediate in the biosynthetic pathway of alazopeptin, a tripeptide having anticancer and antitrypanosomal activities [42]. In a recent study, two distinct lysine 5-hydroxylases, which perform stereoselective 5-hydroxylation of lysine, were discovered [43]. 5-hydroxylysine was identified as one of the metabolic indicators of keloid severity [44]. In this work, we have employed commercially available DL-5-hydroxylysine (5-HL) to probe the elasticity of the active site 5,6-LAM with respect to the perturbation at C5 of the native substrate using various spectroscopic techniques, including EPR spectroscopy and computational analysis.

## 2. Results and Discussion

**Figure 1:**
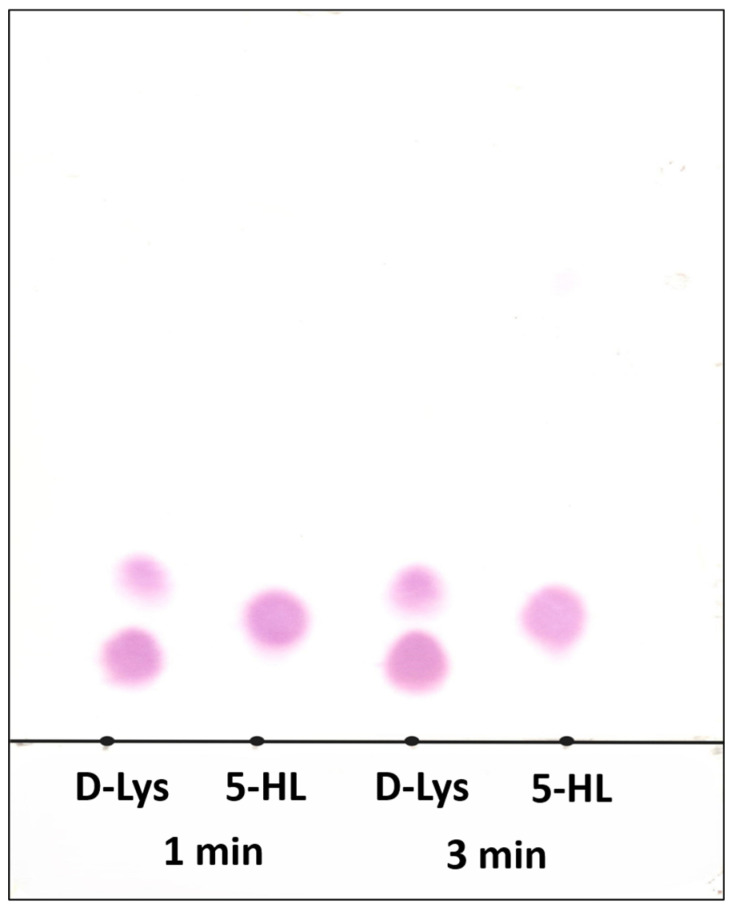
Thin-layer chromatographic (TLC) separation of substrate D-lysine (D-Lys), 5-HL, and product (if any). Lane 1 and Lane 3 correspond to the reactions of D-Lys (lower spots; upper spots correspond to product 2,5-DAH) with 5,6-LAM for 1 and 3 min, respectively. Lane 2 and Lane 4 correspond to 5-HL in the reactions with 5,6-LAM for 1 and 3 min, respectively. Mobile phase: 8:8:3 CHCl_3_/CH_3_OH/12% NH_4_OH mixture. Developer: Ninhydrin (0.2 g/100 mL C_2_H_5_OH).

The reaction of 5-HL and 5,6-LAM does not afford the related product as revealed by the monitoring of reaction by thin layer chromatographic (TLC). TLC of the reaction mixtures was performed by quenching the reaction between 5-HL and 5,6-LAM after 1 min and 3 min. For a comparison, TLC of the reaction mixtures of 5,6-LAM and D-lysine, the natural substrate, with identical conditions was performed alongside (Figure 1). In the case of D-lysine, two distinct spots—the bottom spot corresponding to D-lysine and the top spot corresponding to the product—can be observed for both 1 min and 3 min reaction times. But only one spot corresponding to 5-HL can be observed for both timepoints. This confirms that there is no product formation in the case of 5-HL.

**Figure 2:**
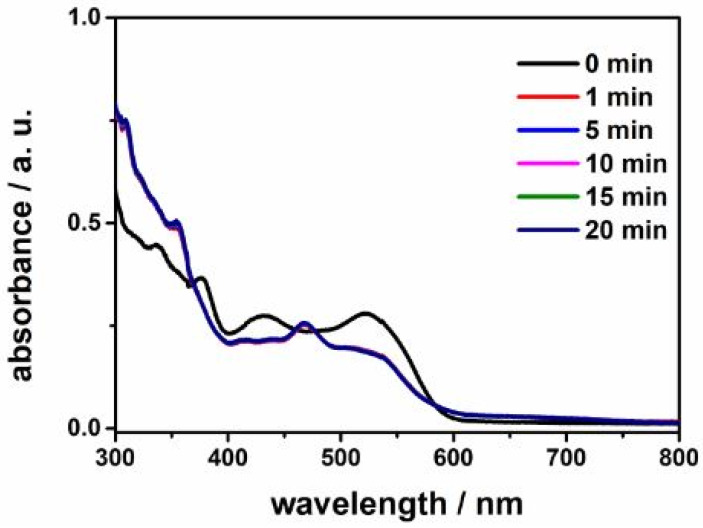
UV–visible spectra in the reaction of 5,6-LAM with 5-HL.

Then we employed UV–visible spectroscopy to find out whether 5-HL is accepted in the active site in the steady-state reaction condition of 5,6-LAM. The UV–Vis spectra (Figure 2) show obvious changes in the spectral features after the addition of 5-HL to the holoenzyme 5,6-LAM containing PLP and dAdoCbl. Attenuation of intensity in the band at 431 nm is assigned to the formation of external aldimine [7]. Decreases in absorptions at 523 nm and 377 nm correspond to the cleavage of the Co–C bond, and the appearances of the new bands at 468 nm and 354 nm are attributable to cob(II)alamin and cob(III)alamin, respectively [24,45]. These spectral changes demonstrate that 5-HL mimics the initial steps of transaldimination and Co–C bond rupture. Therefore, the active site is capable of accommodating the perturbation at the C5 of the substrate introduced by the additional hydroxy group present in 5-HL.

**Figure 3:**
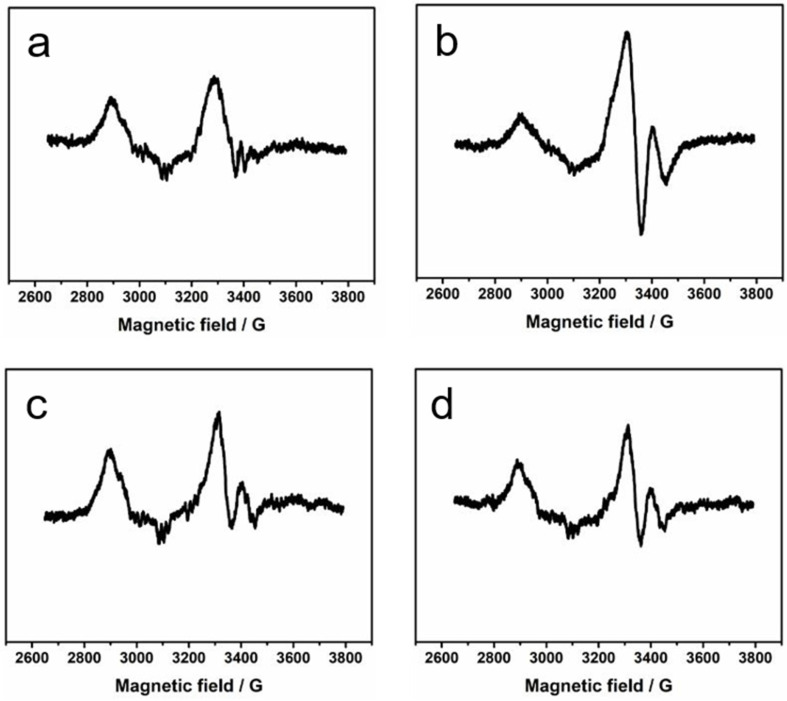
X-band EPR spectra of 5-HL in the reaction with 5,6-LAM after 8 s (**a**), 15 s (**b**), 30 s (**c**), and 45 s (**d**). Experimental conditions: microwave frequency = 9.467 GHz, microwave power = 2 mW, modulation amplitude = 4 G, modulation frequency = 100 kHz, and temperature = 77 K.

**Scheme 1:**
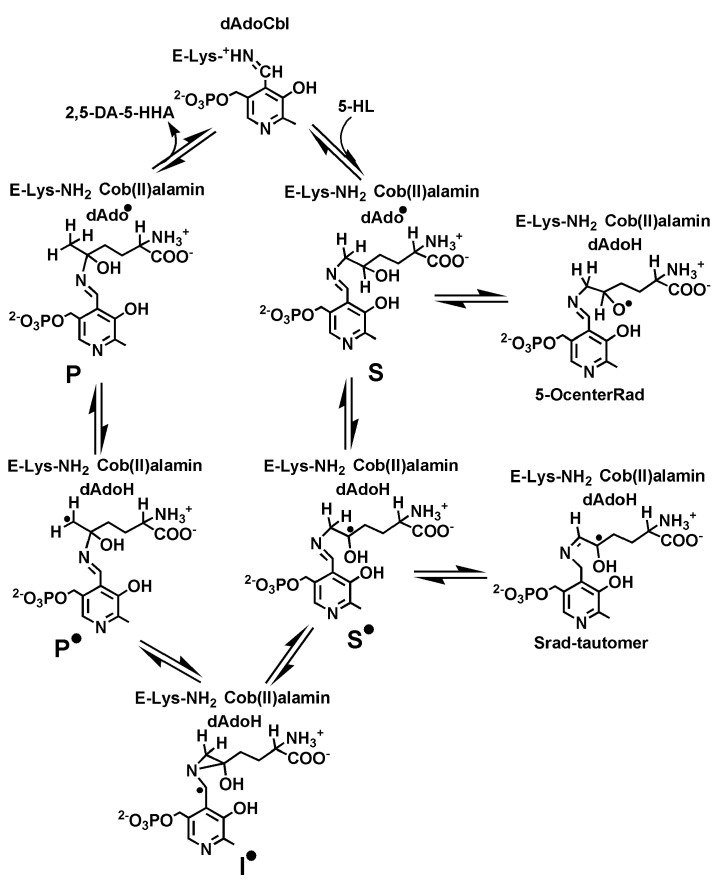
Proposed catalytic cycle and other relevant radical intermediates for the reaction of 5-HL with 5,6-LAM.

At this point, EPR spectroscopy was employed to find out the presence and the nature of paramagnetic species, if any, during the reaction of 5-HL and 5,6-LAM. Figure 3 depicts the EPR spectra of the reactions of 5-HL with 5,6-LAM at four different timepoints—8 s, 15 s, 30 s, and 45 s. A careful visual comparison of all the spectra reveals that all of them contain similar features. Each spectrum comprises two features—a major feature and a very minor feature. The major feature can be attributed to a weakly spin-coupled Co(II) with an organic radical [11,12]. The minor contribution from uncoupled Co(II) can also be noticed. However, the intensity of the major feature attains the highest intensity at the 15 s spectrum, while the intensity of the minor feature remains almost static at all timepoints. In the 15 s spectrum, the minor feature is difficult to detect as it is buried inside the highly intense signal of the major feature. The major feature indicates the cleavage of the Co–C bond to generate Co(II)alamin and dAdo^•^, which then abstract a hydrogen atom from substrate analogue 5-HL-PLP adduct (**S**, Scheme 1) to produce the substrate analogue related radical (**S^•^**, Scheme 1). **S^•^** is transient in nature and may easily transform into other 5-HL-PLP-based radical intermediates (Scheme 1). So, the organic radical detected in this spin-coupled system may or may not be **S^•^**.

**Figure 4:**
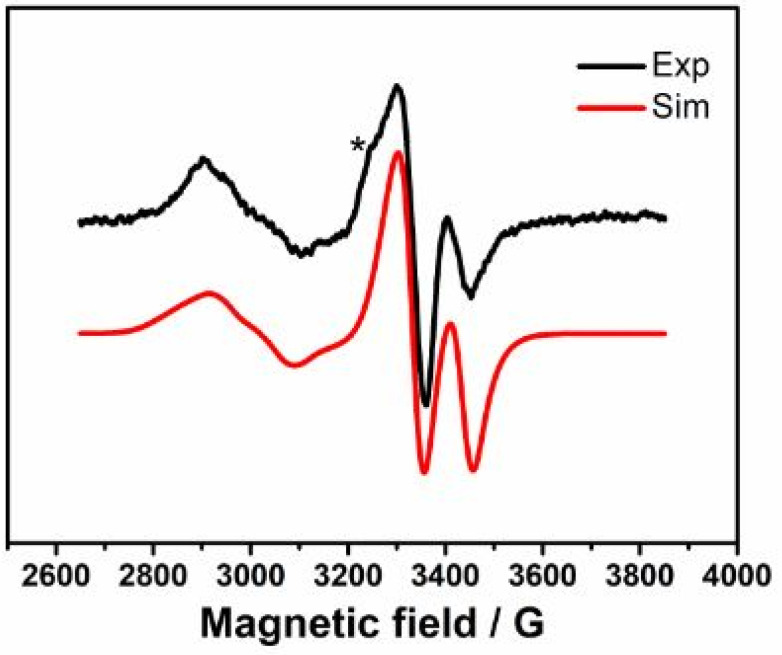
X-band EPR spectrum (black) of 5-HL in the reaction with 5,6-LAM after 15 s. Microwave frequency = 9.467, T = 77 K. Simulated spectrum (red) parameters: g_Co_ = [2.285 2.236 2.010]; g_radical_ = [2.018 2.000 1.996]; Euler angle = [60 10 120]; A_Co_ = [10 7 112] G, A_N_ = 19 G; J = 98 G; D = −19 G, E = 0, Euler angle = [0 42 0]; HStrain = [87 38 151] G; * represents unknown contaminant present in the sample.

We note that a strongly spin-coupled feature was not observed even at 8 s EPR spectrum in the case of 5-HL. This is in contrast to that observed in the reactions of 4-thialysine with 5,6-LAM, D298N, and D298A mutants of 5,6-LAM, respectively. In each case, a strongly spin-coupled spectrum was observed corresponding to a substrate analogue (4-thialysine)-related radical (**S^•^_TL_**) [45,46]. Subsequently, with longer reaction time, this strongly spin-coupled spectrum transforms into the persistent weakly spin-coupled spectrum that is attributable to a tautomer radical (Srad-tautomer_TL_). The absence of a strongly spin-coupled EPR spectra preceding the weakly spin-coupled spectra indicates that the radical observed at 15 s with 5-HL is probably not **S^•^**. Figure 4 depicts the simulation of the EPR spectrum at 15 s. The experimental spectrum can be satisfactorily simulated by ignoring the minor feature that is hidden under the highly intense major feature. The simulation reveals that the major feature corresponds to a weakly spin-coupled system between Co(II) and a 5-HL-PLP-based radical having a zero-field splitting (D) value of −19 G and isotropic exchange coupling constant (J) value of 98 G. The D value of −19 G corresponds to an interspin distance of ~11.7 Å between Co(II) and the 5-HL-PLP-based radical. In contrast, the minor feature, which is visible in the 8 s, 30 s, and 45 s spectra, resembles the uncoupled Co(II) spectrum that was observed when 5,6-LAM was treated with methylhydrazine [47]. The origin of this feature in the case of 5-HL could tentatively be attributed to the binding of one of the four stereoisomers of 5-HL in an unsuitable orientation such that dAdo^•^ is unable to abstract a hydrogen atom from **S**.

**Figure 5:**
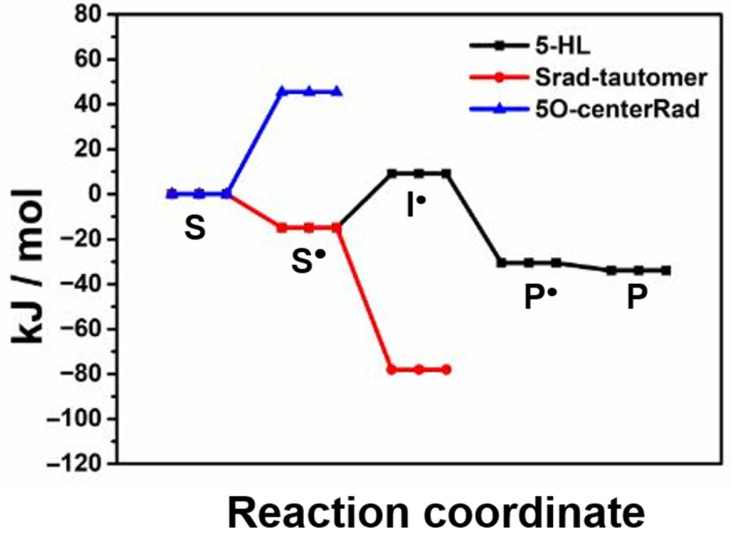
Computed relative energies of various intermediates in the catalytic cycle.

To find out the molecular structure of the 5-HL-PLP-based radical, we have performed density functional theoretical (DFT) calculations following the established methodologies for the relevant intermediates in the proposed catalytic cycle [48,49,50]. Scheme 1 displays the molecular structures of the relevant intermediates in the reaction of 5,6-LAM with 5-HL, which include **S**, **S^•^**, the intermediate radical (**I^•^**), the product analogue-related radical (**P^•^**), and the product analogue-PLP adduct (**P**). Figure 5 displays the relative energies of the pertinent intermediates obtained from DFT calculations in the reaction of 5-HL. These values are compared with the corresponding intermediates in the reaction of lysine and 4-thialysine, respectively, in Table 1. The corresponding substrate-related radical (**S^•^_Lys_**) could not be detected in the reaction of natural substrate lysine. DFT calculations suggested that **S^•^_Lys_** evades the detection by EPR because of negligible accumulation of **S^•^_Lys_** during the catalysis, as it is not stable enough [50]. Interestingly, the authors predicted the detection of **S^•^_TL_** in the reaction of 4-thialysine, as **S^•^_TL_** is highly stabilized by −37.7 kJ/mol (Table 1). Later, **S^•^_TL_** was successfully detected and characterized by EPR spectroscopy [45,46]. Nevertheless, **S^•^_TL_** is transient and tautomerizes into Srad-tautomer_TL_, which is further stabilized by 26.4 kJ/mol than **S^•^_TL_** [45]. A similar energy profile (Table 1) of 5-HL as compared to that of lysine indicates that it should afford the product in the gas phase, as it is comparable with that of lysine. On the basis of similar relative energies of **S^•^** and **S^•^_Lys_**, like in the case of **S^•^_Lys_**, the detection of **S^•^** by EPR is not possible. In contrast to lysine, the catalytic cycle is broken at one stage in the case of 5-HL either by over-stabilization of the intermediate in one of the steps of the native catalytic cycle or due to the transformation to other feasible unproductive pathways. Hence, we have also calculated the relative energies of two other putative radical intermediates which may be generated due to transformations not pertaining to the catalytic cycle: first, an alkoxyl radical (5-OcenterRad, Scheme 1) generated from the abstraction of the hydrogen atom from the 5-OH group of 5-HL; second, a tautomerized radical (Srad-tautomer, Scheme 1) from **S^•^** similar to Srad-tautomer_TL_ that was observed in the case of 4-thialysine [16,45].

The 5-OcenterRad radical is destabilized by 45 kJ/mol and hence very unlikely to accumulate for the EPR detection. On the other hand, the Srad-tautomer is highly stabilized by 78 kJ/mol and may be a plausible candidate for the radical intermediate observed. The Srad-tautomer is generated as a result of the isomerization of **S^•^**. In the case of 4-thialysine, the observation of the Srad-tautomer_TL_ was preceded by the observation of the **S^•^_TL_** [45]. If the radical observed in this work is to be ascribed to the Srad-tautomer, a strongly spin-coupled EPR spectrum corresponding to **S^•^** must be observed prior to the weakly spin-coupled EPR spectra. No such strongly spin-coupled EPR spectra corresponding to the **S^•^** has been observed in the case of 5-HL, and it seems less likely that the radical observed is Srad-tautomer. Interestingly, **P^•^** in Scheme 1 is the most stabilized radical intermediate among the intermediates generated in the normal course of the catalytic cycle. It is also stabilized by 15 kJ/mol as compared to that of lysine. Although in the gas phase it should afford the product, in the enzyme either the steric bulk of the hydroxyl group or unwanted hydrogen bonding between the 5-hydroxyl group and putative active site residues may result in a conformation that blocks the reabstraction of a hydrogen atom from 5′-deoxyadenosine (dAdoH) to give rise to the product and induce 5,6-LAM to a semi-open state. Of course, there is also the probability that a similar situation may arise in the case of the substrate-related radicals. However, in that case one would expect a strongly spin-coupled EPR spectrum similar to that observed in the reaction of 4-thialysine with 5,6-LAM, D298N, and D298A mutants of 5,6-LAM, respectively; instead, only a weakly spin-coupled spectrum has been observed in the case of 5-HL. So, on the basis of the pattern of EPR spectra along with the analysis of relative energies of various putative intermediates, we tentatively assign the observed organic radical to **P^•^** in Scheme 1. Future EPR and ENDOR experiments with synthesized site-directed labelled all four stereoisomers of 5-HL [51,52], like we reported in the case of 4-thialysine [16,46,53,54], are required to determine the structure of the radical unequivocally. Moreover, the experiments with individual stereoisomers should also shed further light on the major and minor features of the EPR spectra of 5-HL. Nevertheless, a comparison of interspin distance obtained from simulation provides new insight into the conformational dynamics of 5,6-LAM. Similar interspin distances of ~11.7 Å between Co(II) and **S^•^**, as in the case of 5-HL, were also observed previously in 5,6-LAM. An interspin distance of 10.3 Å was estimated in the case of the reaction of 4-thialysine and the D298N mutant of 5,6-LAM for 3 min [7]. Srad-tautomer_TL_ observed in the reaction of 4-thialysine and 5,6-LAM is also positioned at ~10.6 Å. An inter-spin distance of ~10–12 Å is in between 5.5 Å and 25 Å, corresponding to the closed and the open states, respectively. We attribute this intermediate positioning of Co(II) and PLP substrate analogue-based organic radical in 5,6-LAM to an adoption of a distinct conformational state that is different from either the open state or the closed state. The observations of this state by two different substrate analogues—in the forms of Srad-tautomer_TL_ with 4-thialysine and **P^•^** with 5-HL—imply the putative existence of an analogous conformational state during the canonical reaction of 5,6-LAM. It is worthwhile to note that both 4-thialysine and 5-HL act as the inhibitors of 5,6-LAM, eventually leading to the suicide inactivation of it. Now, the question arises as to what role this conformational state might play. This conformational state could be visualized as a hidden minor conformation present in the ensemble of differently populated conformational states. Substrate analogues induce the increase in the population of this conformer. As mentioned above, 5,6-LAM undergoes suicide inactivation. It is imperative that it must have an alternative pathway for suicide inactivation rather than the desired course of reaction. We hypothesize that this alternative pathway involves this hidden conformation of 5,6-LAM. A similar open–closed–inactivated conformational triad is also present in voltage-gated sodium channels [55]. Therefore, we assign this conformation to the suicidally -inactivated conformational state of 5,6-LAM. Thus, we have demonstrated an approach of employing simple EPR spectroscopy to uncover the hidden conformations of 5,6-LAM that could be emulated in other radical enzymes. We envisage that X-ray crystallography on crystals of 5,6-LAM obtained with an inhibitor, like in the case of dAdoCbl-dependent enzyme methylmalonyl-CoA mutase [13], would reveal the structure of this hidden conformation. Nonetheless, in the absence of a crystal structure, computational studies following either the approach used in the case of 4,5-OAM [56] or advanced molecular dynamics simulations of the conformational free energy landscape of 5,6-LAM, described by Maria-Solano and coworkers [23], should shed light on the structure of this hidden conformation.

## 3. Materials and Methods

5-HL was purchased from Sigma-Aldrich, St. Louis, MO, USA. The recombinant 5,6-LAM from *Clostridium sticklandii* was expressed in *Escherichia coli* and purified following the procedures described earlier [16,47].

### 3.1. UV–Visible Spectroscopy

25 μM 5,6-LAM preincubated with 25 μM dAdoCbl and 25 μM PLP for 5 min at 37 °C in 100 mM NH_4_-EPPS buffer at pH 8.5 was used as the holoenzyme mixture. The UV–vis spectra were measured at various time intervals (1 min, 5 min, 10 min, 15 min, and 20 min) after the addition of 5-HL (2.5 mM) using a Shimadzu UV-2550 spectrophotometer.

### 3.2. EPR Spectroscopy

5,6-LAM (~0.25 mM) was incubated with 100 mM NH_4_EPPS buffer at pH 8.5, 5 mM dithiothreitol, 0.3 mM dAdoCbl, and 0.3 mM PLP at 37 °C for 5 min. Anaerobic holoenzyme mixture and bulk solution containing substrates were prepared by repeated evacuation and backfilling with argon. The reaction was initiated by the addition of 30 mM 5-HL to the holoenzyme mixture anaerobically. The reaction mixture (~0.2 mL) was allowed to proceed for either 8 s, 15 s, 30 s, or 45 s, and then frozen immediately in liquid nitrogen-chilled isopentane. X-band EPR measurements were performed using a Bruker EMX spectrometer equipped with a TE102 cavity using a cold finger quartz Dewar with temperature maintained at 77 K. All the spectra have similar features. However, the 15 s EPR spectrum has the highest intensity. So, we simulated the major feature of the 15 s spectrum using EasySpin software version 5.2.6 [57]. The EPR spectrum was simulated using a biradical spin-Hamiltonian that includes one-electron spin-Hamiltonians, isotropic exchange coupling “J”, zero field splitting “D”, nuclear hyperfine interaction of low spin Co^2+^ with the ^59^Co nuclear spin (I = 7/2) and with the α-axial ligand ^14^N nuclear spin (I = 1). Other nuclear hyperfine interactions are assumed to contribute to the inhomogeneous linewidth of the EPR features and are neglected in the simulation. Eulerian angles were used to relate all coordinate systems to the principal g-axis of Co^2+^.

### 3.3. Computational Details

Optimized geometries and vibrational frequencies (scaled by 0.9806) [58] were obtained in the gas phase at the B3-LYP level with 6-31G(d,p) basis. Improved relative energies at 0K were calculated at the RMP2/G3MP2Large level. This level of theory has been successfully employed to have insights into dAdoCbl-dependent reactions, including 5,6-LAM [3,7,48,49,50,59,60]. The methyl group and the phosphate handle of PLP were replaced with hydrogen atoms. The N1 of the pyridine ring of PLP is not protonated as suggested by the crystal structure [6] of 5,6-LAM. dAdoH was modelled as 2-methyltetrahydrofuran-3,4-diol. The Gaussian 09 programme [61] suite was used for all the calculations.

## 4. Conclusions

In summary, 5-HL behaves as a mechanism-based inhibitor for 5,6-LAM by locking it in the suicidally-inactivated conformational state while mimicking most of the steps of the catalytic cycle of 5,6-LAM. Here, simple EPR spectroscopic studies are employed to unravel the existence of this distinct hidden conformational state. Nevertheless, structural and computational studies are needed to gain a comprehensive understanding of this distinct conformational state. Additionally, these investigations would shed light on the mechanism by which the enzyme transforms into the suicidally-inactivated conformational state. Our findings would pave the way for the new investigations to link the involvement of the putative distinct conformational state to the suicide inactivation in the radical enzymes including, the dAdoCbl-dependent enzymes. This work heralds the future potential application of EPR spectroscopy in exploring the hidden conformations in the realm of radical enzymes.

## Figures and Tables

**Table 1:** Computed relative energies of various intermediates in the catalytic cycle (n. a. = not available).

	Relative Energy (kJ/mol)		
	5-HL	Lysine ^a^	4-Thialysine ^a^
**S**	0	0	0
**S** **^•^**	−14.9	−10.4	−37.7
**I** **^•^**	9.1	16.9	12.3
**P** **^•^**	−30.6	−15.7	−22.9
**P**	−34.0	−17.1	n. a.
Srad-tautomer	−78.1	n. a.	−64.1 ^b^
5O-centerRad	45.4	n. a.	n. a.

^a^ The values are taken from reference [50]. ^b^ The value is taken from reference [45].

## Data Availability

The original contributions presented in this study are included in the article material. Further inquiries can be directed to the corresponding author(s).

## Abbreviations

The following abbreviations are used in this manuscript:

5,6-LAM.	Lysine 5,6-aminomutase
PLP	Pyridoxal 5′-phosphate
dAdoCbl	5′-Deoxyadenosylcobalamin
EPR	Electron paramagnetic resonance
5-HL	DL-5-hydroxylysine
TLC	Thin-layer chromatographic
DFT	Density functional theory
dAdoH	5′-Deoxyadenosine
